# Surgical Operations in Cancerous Diseases

**Published:** 1844-11

**Authors:** 


*On the utility of Surgical Operations in Cancerous Diseases.* The grand points of this most important surgical question are to determine—1, if it be really true that cancerous disease is primarily of a local nature, and subsequently degenerates into a constitutional malady; and 2, if extirpation, performed at an early period, prevents the occurrence of this degeneration. With the view of elucidating these matters, M. Leroy d'Etiolles has collected the following statistical observations. Of 801 operations, 117 were performed within a twelve month after the first manifestation of the disease.

Of these 117 cases, there were 61 in which the disease had returned at the time when the reports reached me. It is more than probable that this proportion would be found to be still higher, if we knew the actually present state of these cases.

The results of operations for Cancer of the Lip are curious and worthy of notice, in consequence of the differenee in this respect observed in the two sexes. Of 633 cases of Cancer in the male subject, 165 were examples of Cancer of the Lip; of those 114 were treated with the knife—12 with caustics. There were 15 relapses in all; that is, about an eighth of the whole. On the other hand, of 2,148 cases of Cancer in the female, there were only 34 instances of the disease in the lip; of these, 22 were treated by excision; and in seven—nearly a third—there was a return of the disease.

This difference does not hold good of Cancer of the tongue; for then the disease is equally fatal in both sexes. Of nine operations, in which a cancerous tumour of this organ was extirpated, three were performed within one twelve-month after the earliest appearance of the disease. In the other six cases, the patients died, the disease having previously returned.

As respects Cancerous diseases of the Mamma, we find the following data. Of 277 operations, 73 were performed within the last two years: as yet we cannot say positively what are the results. Of the remaining 204 cases, 22 of them proved fatal in the year after the operation, and in 87 others there was a relapse of the disease.

M. Leroy deduces the following conclusions from his researches:

1. Extirpation does not arrest the progress of Cancerous disease.

2. This operation should not be resorted to, as a general method of treatment, except for Cancer of the skin and lips.

3. There is no necessity to extirpate Cancerous disease of other organs, except when an alarming hæmorrhage supervenes.—*Comptes rendus.*

The Academy appointed MM. Roux, Velpeau, and Serres, to report upon this communication of M. Leroy.

*Med. Chir. Rev.*

